# Analyzing Social Vulnerability and ESBL Infection Rates at the Census-Tract Level in Tennessee, 2019–2023

**DOI:** 10.1017/ash.2025.342

**Published:** 2025-09-24

**Authors:** Srilakshmi Velrajan, Darryl Nevels, Melphine Harriott, Daniel Muleta

**Affiliations:** 1 TN Department of Health; 2 Tennessee Department of Health; 3 TN Department of Health HAI/AR Program; 4 Tennessee Department of Health; Michael Norris, State of Tennessee Department of Health

## Abstract

**Background:** There are numerous ways to measure social markers of health. One reliable method for predicting health outcomes is the social vulnerability index (SVI) which assesses multiple themes, including housing insecurity, socioeconomic status, and minority status. As a part of Multi-site Gram Negative Surveillance Initiative (MuGSI), surveillance of Extended-Spectrum Beta-Lactamase (ESBL)-producing Enterobacterales was conducted in four Tennessee counties (Maury, Marshall, Wayne, and Lewis). This study examines the association between social vulnerability and infection rates for ESBL-producing Enterobacterales within the surveillance area. **Method:** ESBL incident cases reported from July 2019 to December 2023 were analyzed. Cases were defined as the first isolation of Escherichia coli, Klebsiella pneumoniae, or Klebsiella oxytoca resistant to at least one extended-spectrum cephalosporin (ceftazidime, cefotaxime or ceftriaxone) and non-resistant to all carbapenem antibiotics from urine or normally sterile sites in residents of the surveillance area within a 30-day period. Pearson correlation analysis was conducted to evaluate the association between SVI scores and ESBL infection rates per 1,000 residents at the census tract level, as well as the four SVI ranking variables (socioeconomic status, household characteristics, racial & ethnic minority status, and housing type & transportation). Analysis was conducted using SAS 9.4. Geospatial analysis in ArcGIS Pro v2.9.7 produced a bivariate choropleth map, illustrating the interaction between SVI and ESBL infection rates. **Result:** From 2019–2023, 2,166 ESBL cases were reported. Cases were 21% male and 79% female, with mean age of 66 years. Incidence rates ranged from 0.19 to 19.5 per 1,000 population. The analysis revealed a significant positive relationship between SVI and tract-level ESBL infection rates. Higher vulnerability scores are associated with higher infection rates, as evidenced by the positive correlation coefficient (&#8477? = 0.38427, &#8477? = 0.0272). Pearson correlation analysis revealed that household type and transportation demonstrated statistically significant positive correlation with ESBL infection rates (&#8477? = 0.431, &#8477? = 0.0121). **Conclusion:**Information from geocoding surveillance data can be used to identify social groups at increased risk of infections with drug resistant pathogens. In this study, ESBL infection rate is significantly associated with SVI. Among the four themes, only household type & transportation status is found to be significantly associated with ESBL infection rates. Further research is needed to understand the role housing plays in the spread of ESBL infection, especially looking at both urban and rural populations. Using SVI scores as a risk assessment tool, infection preventionists and antibiotic stewards can prioritize high risk areas for intervention.

